# An outsider on the Antarctic Peninsula: A new record of the non‐native moth *Plodia interpunctella* (Lepidoptera: Pyralidae)

**DOI:** 10.1002/ece3.10838

**Published:** 2024-02-05

**Authors:** Hugo A. Benitez, Carla Salinas, Jordan Hernández, Tamara Contador Mejías, Sanghee Kim, Claudia S. Maturana, Lorena Rebolledo, Laura M. Pérez, Paulo E. A. S. Câmara, Vinícius Alves Ferreira, Isabel Lobos, Alejandro Piñeiro, Peter Convey

**Affiliations:** ^1^ Millennium Institute Biodiversity of Antarctic and Subantarctic Ecosystems (BASE) Santiago Chile; ^2^ Cape Horn International Center (CHIC) Centro Universitario Cabo de Hornos, Universidad de Magallanes Puerto Williams Chile; ^3^ Laboratorio de Ecología y Morfometría Evolutiva, Centro de Investigación de Estudios Avanzados del Maule Universidad Católica del Maule Talca Chile; ^4^ Departamento Científico Instituto Antártico Chileno Punta Arenas Chile; ^5^ Programa de Doctorado en Salud Ecosistémica, Centro de Investigación de Estudios Avanzados del Maule Universidad Católica del Maule Talca Chile; ^6^ Núcleo Milenio de Salmónidos Invasores (INVASAL) Concepción Chile; ^7^ Division of Life Sciences Korea Polar Research Institute Incheon Korea; ^8^ Departamento de Física, FACI Universidad de Tarapacá Arica Chile; ^9^ Departamento de Botânica Universidade de Brasília Brasília Brazil; ^10^ Agronômica, Laboratório de Diagnóstico Fitossanitário e Consultoria Porto Alegre Brazil; ^11^ British Antarctic Survey (BAS) Natural Environment Research Council Cambridge UK; ^12^ Department of Zoology University of Johannesburg Auckland Park South Africa

**Keywords:** Antarctica, Indian meal moth, non‐native species, research stations, Yelcho Station

## Abstract

We report the first record of the microlepidopteran *Plodia interpunctella* beyond the South Shetland Islands at the Chilean Yelcho scientific station (64°52′33.1428″ S; 63°35′1.9572″ W), Doumer Island, close to the west coast of the Antarctic Peninsula. It is notable that *P. interpunctella*, a globally distributed stored product pest species, exhibits a remarkable capacity for prolonged viability within food storage facilities. The dual challenges of food transportation and storage in the context of Antarctica's challenging operational conditions may have facilitated *P. interpunctella'*s initial arrival to the Antarctic region. Non‐perishable food items, such as grains, flour and rice, provide practical options for the bulk food transportation and storage required in the long‐term operation of Antarctic research stations. The presence of *P. interpunctella* in Antarctica, even if restricted to synanthropic environments within buildings, is a clear threat to Antarctic biodiversity, not only through being an invasive species itself but also as a potential vector for other non‐native species (bacteria, acari, between others.), which could carry diseases to the native species.

## Graphical abstract

This text reports the discovery of the microlepidopteran *Plodia interpunctella* at the Chilean Yelcho scientific station on Doumer Island, near the west coast of the Antarctic Peninsula. *Plodia interpunctella* is a global pest known for surviving in stored food for extended periods. The text suggests that the insect may have initially arrived in Antarctica due to the challenges of food transportation and storage in the harsh Antarctic conditions. Non‐perishable food items like grains and flour are commonly transported to Antarctic research stations, potentially aiding the insect's introduction. The presence of *P. interpunctella* in Antarctica, even within buildings, poses a threat to Antarctic biodiversity as an invasive species and a potential vector for other non‐native species.

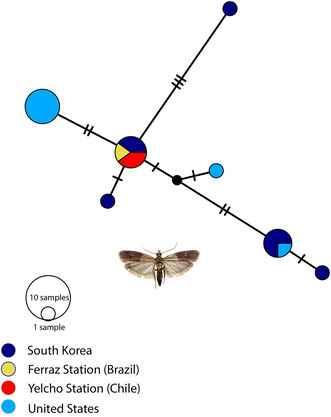

## 1 INTRODUCTION

Increasing globalization and economic development (Menchaca Dávila & Alvarado Michi, 2011) have eroded geographical barriers, with significant and even transformative impacts on natural ecosystems (Espinosa, 2018). Antarctica is one of the few pristine environments remaining on the planet, due to its physical isolation and extreme climatic conditions (low temperatures, lack freshwater, strong winds, short growing season and limited ice‐free areas), which act as barriers to the natural colonization and establishment of organisms (Convey et al., 2014; Duffy et al., 2017; Hughes & Convey, 2010). The terrestrial biodiversity of the Antarctic continent is characterized by a high degree of endemism (Chown et al., 2015; Chown & Convey, 2007; Convey et al., 2020; Pugh & Convey, 2008) and low overall species diversity (Convey & Stevens, 2007). Mosses and lichens dominate ice‐free terrestrial areas, while the invertebrate fauna is limited to small arthropods and microinvertebrates (Convey & Biersma, 2024; Hughes & Pertierra, 2016). There are only two species of native insects, both chironomid midges, Antarctica's only flying insect, *Parochlus steinenii*, and the wingless *Belgica antarctica*, the latter endemic to the Antarctic Peninsula and South Shetland Islands (Chown & Convey, 2016; Contador et al., 2020).

In recent decades, human activity on the continent has increased (Contador et al., 2023; Tejedo et al., 2022; Tin et al., 2009), with scientific activities and tourism growing rapidly other than during the short hiatus imposed by the global COVID‐19 pandemic (Hughes & Convey, 2020). At the same time, the Antarctic Peninsula region has faced particularly rapid regional climate change (Turner et al., 2009), with future scenarios currently being relatively unconstrained (Siegert et al., 2019). Acting in synergy, these factors have facilitated the anthropogenically assisted introduction of non‐native terrestrial species into the area of Antarctic Treaty governance (Hughes et al., 2015; Pertierra et al., 2016). Biological invasions are arguably the greatest contemporary threat to Antarctic ecosystems and biodiversity (Convey & Peck, 2019), in addition to being recognized as one of the five major causes of biodiversity loss globally, acting in multiple and generally unknown ways and affecting multiple levels of ecosystem organization (Tellería, 2013). The presence of species such as the dipteran *Trichocera maculipennis* (Remedios‐De León et al., 2021; Volonterio et al., 2013), or the recent first record of the Indian meal moth *Plodia interpunctella* within the Antarctic Treaty Area in 2021, at the Brazilian Commandante Ferraz research station in Admiralty Bay on King George Island (South Shetland Islands; (Câmara et al., 2022)) and a previous sighting at the Korean King Sejong station in 2017 (pers. obs. Chanh‐Young Choi and Yu‐Min Kim), are among the recent examples of anthropogenic species introductions into Antarctica. *Plodia interpunctella* was previously similarly reported as an introduction to the King Edward Point research station on the milder sub‐Antarctic island of South Georgia in 2000 (Convey, 2005).

**FIGURE 1:**
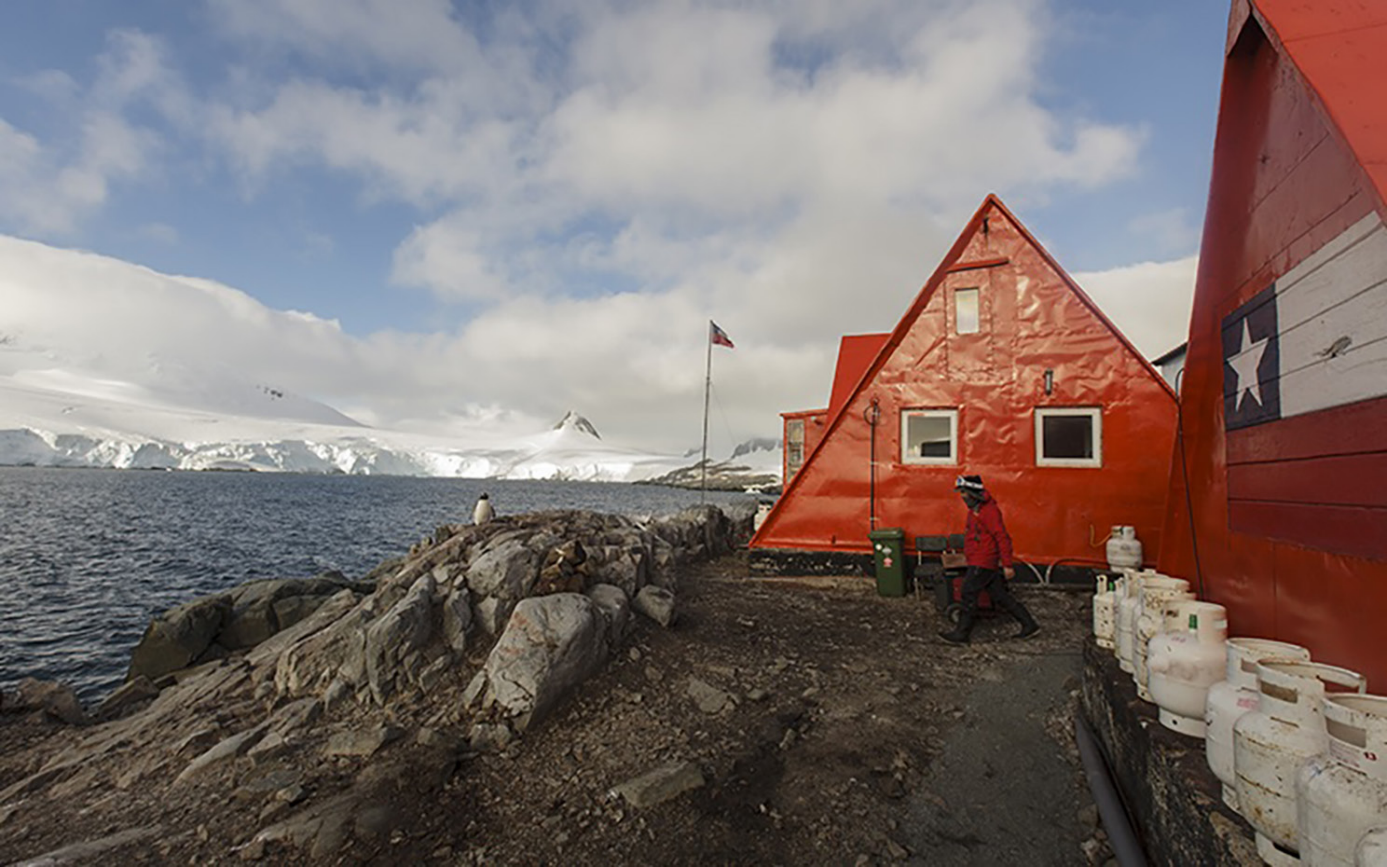
Yelcho Station on the shore of South Bay, Doumer Island, west of the Antarctic Peninsula coast.

The Instituto Antártico Chileno (INACH) manages three stations supporting research in Antarctica: Professor Julio Escudero Station (King George Island, 62°12′57″ S; 58°57′35″ W), Dr. Guillermo Mann Station (Livingston Island, 62°27′0″ S; 60°47′0″ W) and Yelcho Station. The latter is located at South Bay, Doumer Island (64°52′55″ S; 63°35′03″ W). This article reports the presence of the microlepidopteran, *Plodia interpunctella* (Pyralidae), at Yelcho Station, including confirmation of identity through DNA barcoding, providing the first record of the species beyond the South Shetland Islands in the Antarctic Peninsula region (Figure 1).

## 2 MATERIALS AND METHODS

**FIGURE 2:**
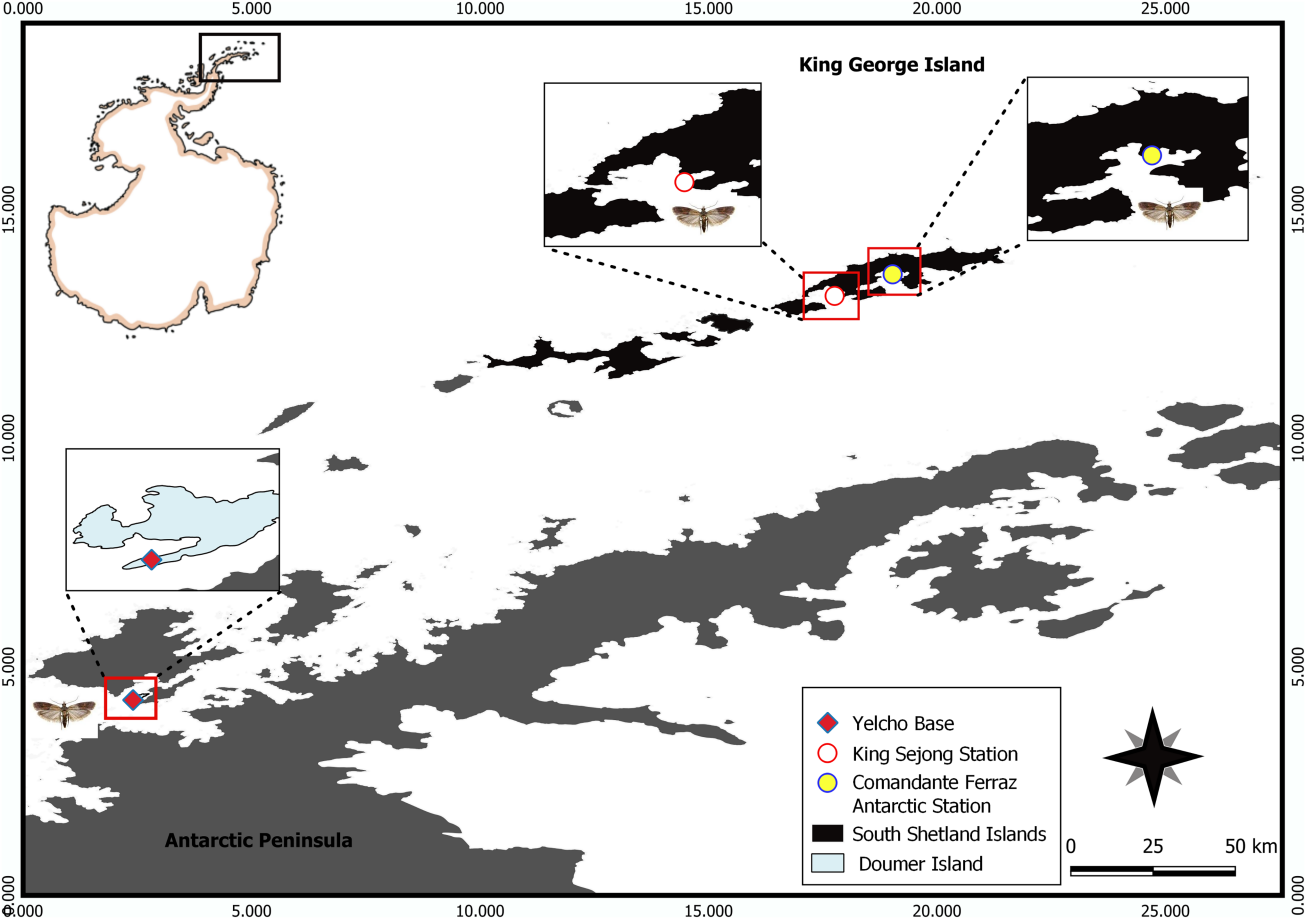
Map showing the three station locations at which *Plodia interpunctella* has been reported in Antarctica.

In February 2022, two specimens of *Plodia interpunctella* were observed flying on different days (February 22 and 24, 2023) and collected on the first and second floors of Yelcho Station (Figure 2). The collected specimens were stored in 95% ethanol and frozen for genetic analyses.

Moth legs were removed to extract DNA using the salting‐out method (Aljanabi & Martinez, 1997). The mitochondrial cytochrome c oxidase I (cox1) gene was amplified using universal primers LCO1490 and HCO2109 in a total of 30 μL of mix containing PCR buffer 1× (200 mM Tris–HCL pH 8.4, 500 mM KCl), MgCl_2_ 2 mM, dNTPs 0.16 mM, Forward and Reverse primers 0.1 μM, Taq DNA Polymerase (Invitrogen™) 0.03 U/μL and DNA 1 ng/μL (Folmer et al., 1994).

Thermal cycling parameters for PCR conditions were: initial denaturation step at 94°C for 3 min, followed by 35 cycles at 95°C for 1 min, 50°C for 45 s (annealing temperature), and 72°C for 30 s, and a final extension of 10 min at 72°C. PCR products were purified and bidirectionally sequenced by Macrogen, Inc. using an automated sequencer ABI3730x1. Sequences were edited and aligned using the ClustalW option of BioEdit v7.0.5 (Hall, 1999). Sequences were inspected, assembled and edited using Geneious software 10.2.2 (Kearse et al., 2012). Finally, genealogical relationships were estimated by constructing a median‐joining haplotype network (Bandelt et al., 1999) using PopArt (http://popart.otago.ac.nz).

## 3 RESULTS

The two specimens observed were collected in the station's working area on the second floor and in the station dining room adjacent to the kitchen on the first floor.

**FIGURE 3:**
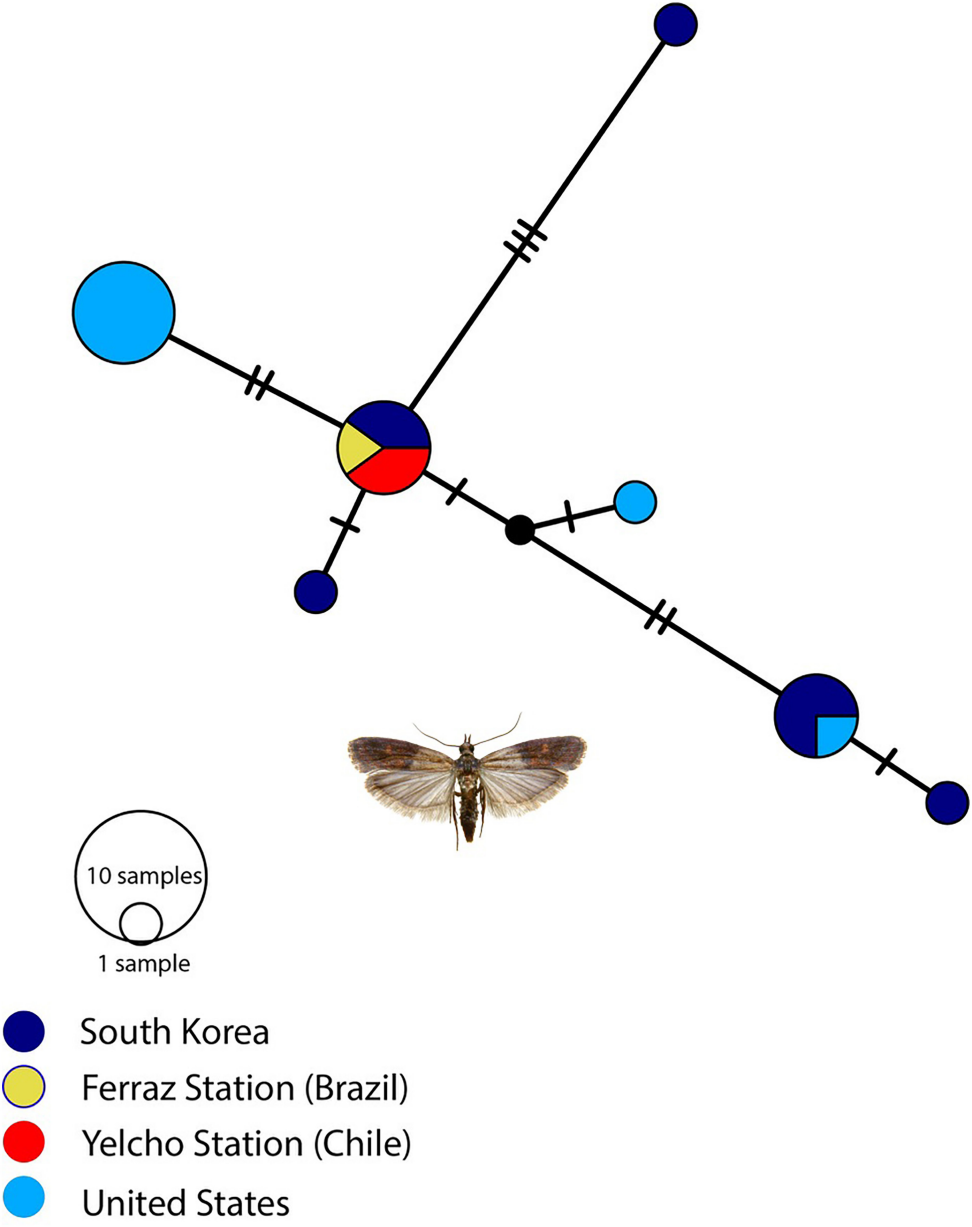
Haplotype network for *Plodia interpunctella*, including mtDNA cox1 sequences from two Antarctic research stations, Commandante Ferraz, Brazil, King George Island (*n* = 1), and Yelcho, Chile, Doumer Island, western Antarctic Peninsula (*n* = 2) and sequences from the United States of America (*n* = 8) and South Korea (*n* = 8). Circle sizes are proportional to the frequency of the haplotype in the entire sample, as shown in the key on the right. The color key indicates the site of collection.

Our molecular analyses included a total of 19 sequences. Three were obtained in the present study, from the specimens collected at Yelcho Station (*n* = 2) and previously from Ferraz Station (*n* = 1, collection described in (Câmara et al., 2022)), combined with 16 sequences obtained from GenBank originating from the South Korean Local populations (*n* = 8) and the United States Local populations (*n* = 8; for accession numbers see Table A1). The final alignment consisted of 593 bp and did not include insertion/deletion or stop codons. Sequences obtained from the specimens from Yelcho Station had a 100% match to the *P. interpunctella* published genome (Wu et al., 2020). Additionally, the cox1 median‐joining network identified one shared haplotype among the three Antarctic research stations from which the species has now been recorded (Figure 3), possibly suggesting a common population history among these locations across the maritime Antarctic region.

## 4 DISCUSSION

The Indian meal moth holds significant economic importance as a pest of stored products, and is now widely distributed across all continents except Antarctica (Mohandass et al., 2007; Rees, 2007). It is often found in synanthropic habitats involved in food storage and is recognized as a pest of a diverse range of products, including grains, flour, nuts, cereals and processed foods (Mohandass et al., 2007). While there is no evidence of any capability of *P. interpunctella* to migrate independently or disperse over long continental distances, instances have been documented where the species has been discovered in infested products during commercial ocean freight transport (Schulten & Roorda, 1984).

*Plodia interpunctella* is a pest that can survive for very long periods in stored food (Choi et al., 2017). The challenge presented by its viability in both food transfer and storage adds to the logistical difficulties inherent in supplying and storing food for Antarctic research stations. Non‐perishable foods, such as grains, flour or rice, are particularly suitable for provisioning stations in Antarctica and are imported in large quantities by operators. Nevertheless, and despite the widespread synanthropic presence of the species globally, it is only in the last 2 years that specimens have been formally recorded in Antarctic research stations (Câmara et al., 2022; current study).

While *P. interpunctella* has only been recorded in synanthropic locations within research stations in Antarctica, and likely has neither the physiological capability to survive (Câmara et al., 2022) or suitable food source in the natural Antarctic environment, this third record of its presence again signals a very clear warning of the risks of inadvertent human‐assisted transfer of non‐native organisms to Antarctica if strict biosecurity controls are not in place and applied. A proportion of such transfers are likely to be suitably adapted to survive and establish in the Antarctic environment, as already clearly demonstrated by the northern boreal trichocerid fly, *Trichocera maculipennis*, on King George Island (Remedios‐De León et al., 2021) and the chironomid, *Eretmoptera murphyi*, on Signy Island (Bartlett et al., 2020). Such species may bring functions that are poorly or not currently represented in Antarctic ecosystems, potentially acting as ecosystem engineers and fundamentally changing ecosystem processes (Bartlett et al., 2023). Introductions such as that of *P. interpunctella* may also generate further threats, as they may be associated with commensal, symbiotic or parasitic species. For instance, it is known that *P. interpunctella* is a host for the parasitoid species, *Habrobracon hebetor* (Hymenoptera; Hasan et al., 2022). While such a parasitoid may seem unlikely to find a suitable host in the native Antarctic terrestrial invertebrate fauna, there is no knowledge of the susceptibility of the two native chironomid midges to any parasites, with none present in Antarctica. The introduction of a parasite that could transfer to the endemic *Belgica antarctica* could be disastrous for this species, while any parasitoid introductions to the milder sub‐Antarctic islands would have access to a considerably wider range of native and often endemic invertebrates. Studies of the microbiome of the introduced *E. murphyi* on Signy Island have also reported association with specific fungi (Bridge & Denton, 2007), flagging the possibility of inadvertent introduction and changes to the native microbial communities (Bridge & Hughes, 2010; Cowan et al., 2011). Therefore, the presence of *P. interpunctella* in Antarctica suggests a clear threat, not only as an invasive species itself but also as a vector for other non‐native species, particularly in the face on global climate change.

## AUTHOR CONTRIBUTIONS

**Hugo A. Benitez:** Conceptualization (equal); formal analysis (equal); funding acquisition (equal); investigation (equal); methodology (equal); project administration (equal); resources (equal); software (equal); validation (equal); writing – original draft (equal); writing – review and editing (equal). **Carla Salinas:** Conceptualization (equal); data curation (equal); funding acquisition (equal); project administration (equal); validation (equal); visualization (equal); writing – review and editing (equal). **Jordan Hernández:** Conceptualization (equal); investigation (equal); resources (equal); visualization (equal); writing – original draft (equal). **Tamara Contador Mejías:** Conceptualization (equal); investigation (equal); resources (equal); software (equal); writing – original draft (equal); writing – review and editing (equal). **Sanghee Kim:** Conceptualization (lead); data curation (supporting); investigation (supporting); methodology (supporting). **Claudia S. Maturana:** Conceptualization (equal); data curation (equal); investigation (equal); methodology (equal); software (equal); writing – original draft (equal); writing – review and editing (equal). **Lorena Rebolledo:** Conceptualization (equal); funding acquisition (equal); investigation (equal); project administration (equal); resources (equal); writing – review and editing (equal). **Laura M. Pérez:** Conceptualization (equal); resources (equal); validation (equal); visualization (equal); writing – review and editing (equal). **Paulo E. A. S. Câmara:** Data curation (equal); investigation (equal); software (equal); visualization (equal); writing – review and editing (equal). **Vinícius Alves Ferreira:** Data curation (equal); methodology (equal); validation (equal); writing – review and editing (equal). **Alejandro Piñeiro:** Conceptualization (equal); resources (equal); validation (equal); visualization (equal). **Isabel Lobos:** Conceptualization (equal); resources (equal); validation (equal); visualization (equal). **Peter Convey:** Conceptualization (equal); investigation (equal); resources (equal); validation (equal); writing – original draft (equal); writing – review and editing (equal).

## CONFLICT OF INTEREST STATEMENT

The authors declare no conflict of interest.

## Data Availability

All the genetic data is available at GenBank using the accession number provided in Table A1.

## APPENDIX A

**TABLE A1:** List of sample numbers, collections sites and GenBank accession numbers of cox1 sequences used in this study.

Name	*N*	Collection site	Cox1 accession number
*Plodia interpunctella*, CL	2	Yelcho Research Station, Doumer Island, West Antarctic Peninsula	OR923475 OR923476
*Plodia interpunctella*, BR	1	Ferraz Research Station, King George Island	OR923474
*Plodia interpunctella*, USA	8	n/a (International Barcoding Program)	GU800020, GU800289 GU800968, GU801264, GU801265, GU801375 GU801343
*Plodia interpunctella*, SK	8	Seul, South Korea	KC407753 KC407751 KC407747 KC407752 KC407750 KC407748 KC407746 JQ350723

*Note*: *N*, sample size.
